# Understanding human queuing behaviour at exits: an empirical study

**DOI:** 10.1098/rsos.160896

**Published:** 2017-01-25

**Authors:** A. U. Kemloh Wagoum, A. Tordeux, W. Liao

**Affiliations:** 1 Jülich Supercomputing Centre, Forschungszentrum Jülich, Jülich, Germany; 2 Computer Simulation for Fire Safety and Pedestrian Traffic, University of Wuppertal, Wuppertal, Germany

**Keywords:** pedestrian dynamics, exit choice, load balancing, modelling and simulation

## Abstract

The choice of the exit to egress from a facility plays a fundamental role in pedestrian modelling and simulation. Yet, empirical evidence for backing up simulation is scarce. In this contribution, we present three new groups of experiments that we conducted in different geometries. We varied parameters such as the width of the doors, the initial location and number of pedestrians which in turn affected their perception of the environment. We extracted and analysed relevant indicators such as distance to the exits and density levels. The results put in evidence the fact that pedestrians use time-dependent information to optimize their exit choice, and that, in congested states, a load balancing over the exits occurs. We propose a minimal modelling approach that covers those situations, especially the cases where the geometry does not show a symmetrical configuration. Most of the models try to achieve the load balancing by simulating the system and solving optimization problems. We show statistically and by simulation that a linear model based on the distance to the exits and the density levels around the exit can be an efficient dynamical alternative.

## 1. Introduction

Humans are confronted on a daily basis with a route choice problematic. It happens at large scales such as choosing a different highway to reach a destination or at smaller scales such as picking the right queue at the vending machines or choosing between the right and the left exit when going out of a shopping mall. The route choice is based on subjective attributes such as the familiarity with the location or the experience gathered under similar conditions. Persons familiar with a location might have a preferred route or might avoid some others. The route choice is also based on objective attributes such as the length of the route or the estimated travel time. To calibrate or validate models, empirical data are needed. Field studies are often difficult to understand because the motivation plays an important role. In organized and controlled experiments, we can fix some degrees of freedoms. For instance, all participants can be instructed to leave the room.

In this paper, 16 pedestrian laboratory experiments addressing standard issues in pedestrians’ exit choice are presented. The participants were asked to leave a room presenting several equivalent possible issues. Corridor, square and corner geometries were tested. The initial density levels were sufficiently high to observe congestion at the exits. The results show that load balancing occurs. Such a feature appears in all the experiments we carried out. It confirms that, in a normal situation, the pedestrians choose dynamically the exit in order to minimize their travel times. Then, a minimal model based on the distance to the exits and on the density level in the vicinity of the exits is calibrated and analysed. Most of the models try to achieve load balancing by simulating the system and solving optimization problems. We highlight statistically and by simulation that the minimal model including the density level around the exits can be an efficient dynamical alternative for the modelling of the exit choice.

The second section of this contribution presents related works. The third section describes the experiments and the extraction of features such as trajectories and exit usage. The models for the exit choice and their calibration are described in the fourth section. Validation of the models with simulation are proposed in the fifth section. The last section summarizes the content of the article and provides concluding remarks.

## 2. Related works

Data gathering for the exit choice of pedestrians is performed in real-world [1–4], as well as in virtual environments [5–7]. Participants might behave differently in the virtual environments where the perception is different. However, we observe in both cases that pedestrians are able to dynamically optimize their travel time by choosing adequate exits. In the models, the choice of the exit corresponds to the tactical level of the pedestrian behaviour. Early works consider the shortest path as an adequate solution for uncongested situations [8]. For congested states, the closest exit, if it is congested, may not be the one minimizing the travel time. Therefore, most of the models are based on the distance to the exit and travel time (see e.g. [9–13]). Other factors are also used, such as route preference [4], density level around the exits [1,2], socio-economic factors [7], type of behaviours (egoistic/cooperative, see [3]), or the presence of smoke, the visibility, the herding tendency or again the *faster-is-slower* effect in the case of emergency [4,14–16]. Several types of modelling are developed. Some of them use log-IT or Prob-IT statistical models [4,5,7,11,17]. Some others are based on notions from game theory of pedestrian rationality and objective function [9,10]. While iterative methods such as the Metropolis algorithm or neural networks allow to reach user or system optima by minimizing individual travel time or marginal cost [2,13].

The estimation of travel times in congested situations is a complex problem. Such procedure is realized in general by using simulation of an operational pedestrian model. The coupling to simulation makes the use of the exit model a hard task in terms of computation effort. Yet, there exist strong correlations between the travel time and the density level. They are a consequence of the characteristic fundamental relationship between the flow and the density, that is well established in the literature of traffic theory (see e.g. [18]). Some recent dynamical models are based, among other parameters, on the density levels in the vicinity of the exits (see [1,2,4]). In such models, the density substitutes the travel time. The density levels are simple to measure and, in contrast to the travel time, do not require simulation of the system to be estimated. This makes the density-based models easier to implement than equilibrium-based models.

## 3. Description of the experiments

The experiments were performed within the framework of BaSiGo [19] which aims to understand the behaviour of large crowds in specific situations. The configurations were built with square modules higher than 2.0 m. The participants were arranged in holding areas before the start of the experiments. After an acoustic signal, they were asked to pass through the geometry as quickly as possible but without running or pushing. To guarantee precise measurements, the experiments were recorded by a camera grid of six by four cameras mounted 7.5 m above the floor. Each camera has a resolution of 1280×1024 pixel and a frame rate of 16 fps. Besides, an additional camera to measure the heights of the participants and a fish-eye camera to overlook the entire experimental area were placed. More information about the measurements and extraction of the trajectories is given in [20].

Three groups of experiments and 16 runs addressing standard issues in pedestrian route choice were carried out. In the first group, the participant had an overview of the situation: they entered a room and were faced with two exits to choose. In the second group, the participants started from a square room with four exits. In the third group of experiments, the scene is unclear and there is a visibility factor. The exit number and their position are unknown from the starting location. Such experiments are useful to understand short-term decisions taking in human behaviour under different conditions. They were performed with a total of 138 participants. To avoid and eliminate learning effects, each person participates in one specific experiment set-up only once (excepted for experiment C, run 3).

The data analysis was carried out using the programming tools Python [21] and R [22]. The densities are measured using the Voronoi tessellation method introduced in [23] which is more precise than classical discrete methods.

### 3.1. Experiment A

**Figure 1.**
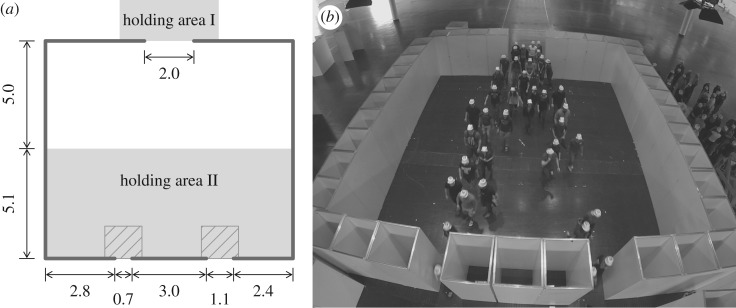
Experiment A—set-up and snapshot. The unit of the sketch is metre. In the first runs, the participants started from the holding area I. For subsequent runs, people were placed in both holding areas. The density was measured in the ridged areas. (*a*) Experiment configuration and (*b*) experiment snapshot.

The first experiment was conceived to understand the exit choice in clear situations. The set-up of the experiment A is presented in figure 1. It consists of a 10 m wide square room with two frontal exits (width of 70 cm and 110 cm). The participants entered the room from the 2 m wide entry situated opposite the exits. Ten experiment runs were performed with the following number of participants: 40, 40, 40, 18, 138, 40, 40, 40, 18 and 138. The participants in the runs with 40 and 18 pedestrians started from the holding area I. In the runs with 138 participants, 90 of them started from the waiting area II and the remaining from the area I with a delay of few seconds.

**Figure 2.**
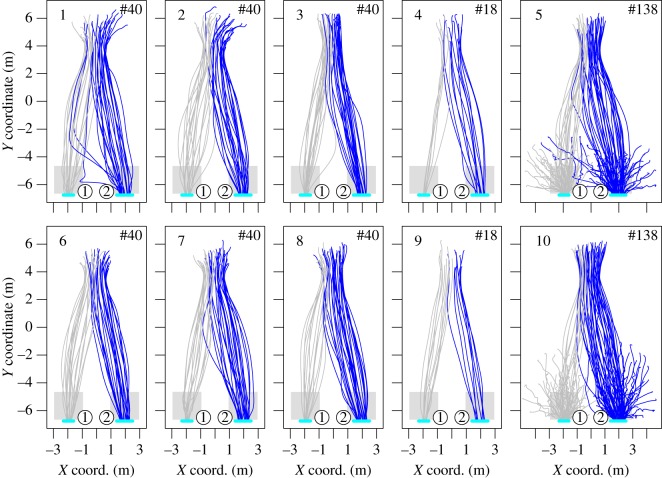
Experiment A—pedestrian trajectories. The pedestrians were moving from the top to the bottom. The colour matches the exit choice. In runs 5 and 10, some participants started from the room. In all other runs, the participants were held in the area outside the geometry.

**Figure 3.**
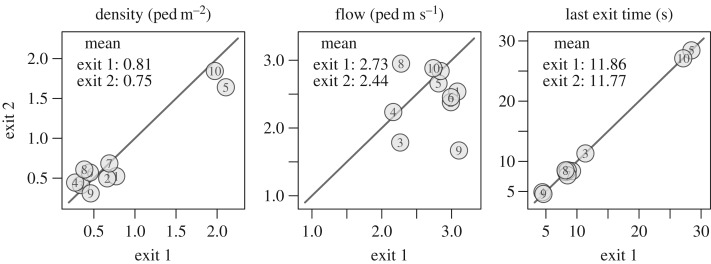
Experiment A—mean performances. The numbers in circles identify the run. The densities observed are similar, except for runs 5 and 10, where we observe congestion at the exits. The last passing times over the exits are approximately equal (load balancing).

The pedestrians’ trajectories are plotted in figure 2 while the mean density, specific flow, and last exit time are given in figure 3. The density is measured in the rectangular areas in front of the exits and the flow is measured when the density is strictly positive. Even if the two exit widths differ, we observe comparable density and specific flow by exit, and a load balancing occurs (the last exit times are the same over the exits). Participants seem to choose their exit after entering the room. Yet, some pedestrians adjust their initial choice (see especially runs 1, 3, 5, 7 and 10). This is a proof of time-dependant information used by the participants.

### 3.2. Experiment B

**Figure 4.**
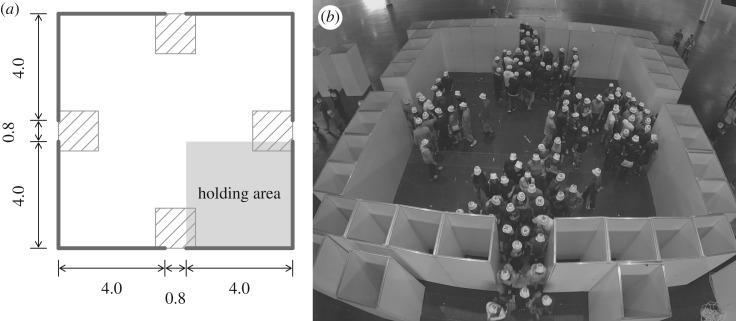
Experiment B—set-up and snapshot. The units of the sketch are metres. The participants had to choose one of the four exits. The density was measured in the ridged areas. (*a*) Experiment configuration and (*b*) experiment snapshot.

The second experiment adds more complexity to the scene. The participants started from one of the corners of a square room and had to choose between four exits that were 80 cm wide and located in the middle of each face of the room. The set-up for the experiment B is presented in figure 4. We performed three runs with (40, 38, 36) participants.

**Figure 5.**
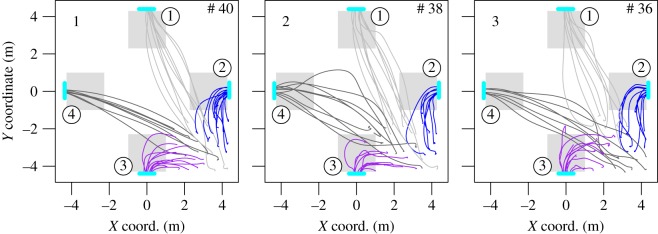
Experiment B—pedestrian trajectories. The pedestrians started in the lower right corner and had to choose between one of the four exits. The colour of the trajectories matches the exit selection. The grey squares near the exits are the measurement areas for the density.

**Figure 6.**
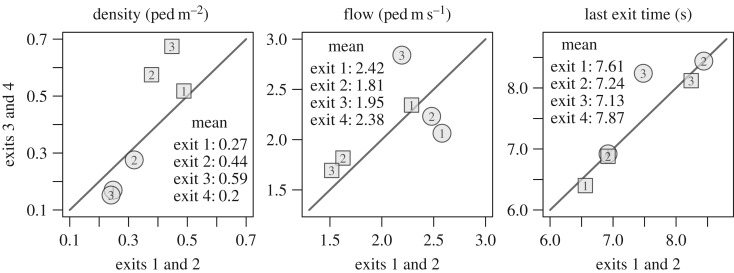
Experiment B—mean performances. The numbers identify the run. The circles correspond to exits 2 and 3 while the squares correspond to exits 1 and 4. Here again, the last passing time is roughly the same for the runs and the overall egress time is optimized.

The pedestrians’ trajectories are plotted in figure 5. The mean density, specific flow and last exit time are given in figure 6. Exits 1 and 2 are grouped on the *X*-axis while exits 3 and 4 are grouped on the *Y*-axis. Such representation allows to compare exit 1 with exit 4 and exit 2 with exit 3, which have similar characteristics (exits 1 and 4 are the farthest ones from the initial positions, while exits 2 and 3 are the closest ones). Here, the compromise between distance and time is clearly visible. All the exits are used, even if two of them are on the opposite side of the room from the initial pedestrian positions. A discrepancy of around 2 s is observed for the evacuation time (figure 6, right panel). This time corresponds approximately to the time needed by a participant to go from one exit to another one (the exits are spaced by around 5 m). This characteristic shows that the participants initially chose an exit and only changed for a freer one if the time won was bigger than the time necessary to reach it. Therefore, the discrepancy is due to the spacial configuration of the experiment and to the initial configuration. Modulo this, the load balancing over the exits occurs again.

### 3.3. Experiment C

**Figure 7.**
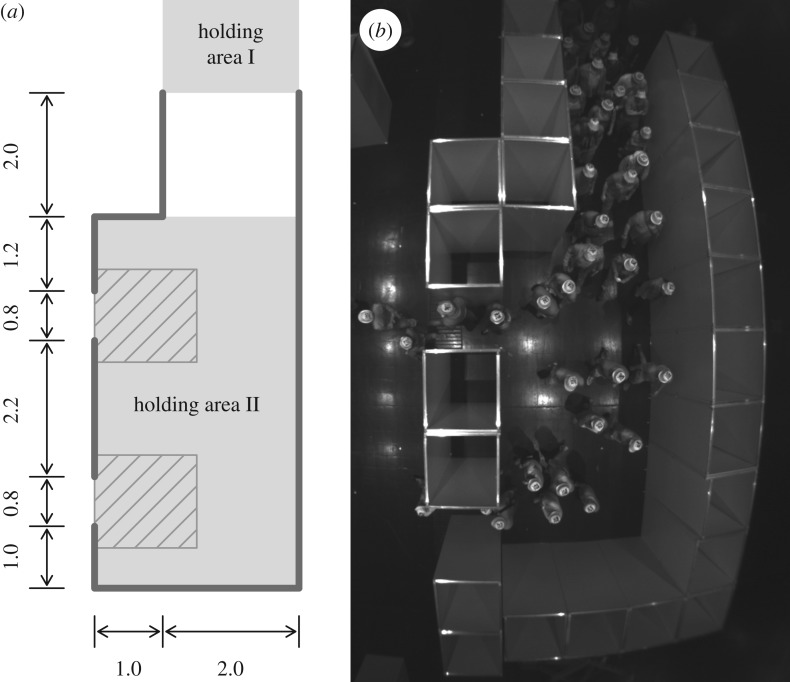
Experiment C—set-up and snapshot. The units of the sketch are metres. In the two first runs, the participants started from holding area I. For the last run, they were placed in both holding areas I and II. The density is measured in the ridged areas. (*a*) Experiment configuration and (*b*) experiment snapshot.

**Figure 8.**
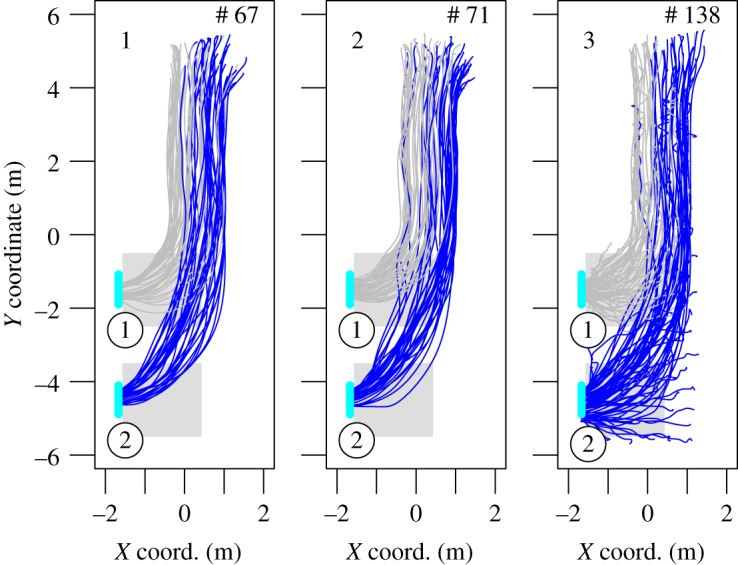
Experiment C—pedestrian trajectories. The pedestrians walked from top to bottom along a corridor and had to choose between two exits. The colour of the trajectories matches the exit selection.

**Figure 9.**
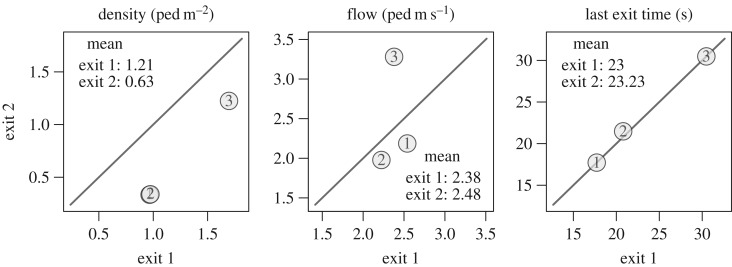
Experiment C—mean performances. The numbers identify the run. Even if the flow and the density can differ from one exit to the other, the last passing time is almost the same over the exits (load balancing).

In the last experiment, the pedestrians walked down a corridor before choosing between two exits in a connected room. While in the corridor there was no possibility to know the number of exits and their position. The set-up of experiment C is presented in figure 7. The room at the end of the corridor had two lateral exits on the same side (exit width 80 cm). We performed three experiment runs with 67, 71 and 138 participants. In the first two runs, the participants started from holding area I, which is in the corridor. In the last run, two groups of 90 and 48 participants were built. They started from holding areas I and II, respectively. Here, the participants of the last run had already performed the experiment once and a learning effect should not be excluded. The pedestrian trajectories are plotted in figure 8 while the mean density, specific flow and last exit time are given in figure 9. We also observe in this situation a load balancing over the different exits.

The empirical results show that the exit chosen by a pedestrian is not always the one minimizing the distance. Each pedestrian is able to estimate and minimize his/her exit time, even if congestion occurs. Consequently, the pedestrians share equitably over the exits available and a load balancing occurs. By doing so, the global exit times are optimized.

## 4. Exit choice models

In this section, we develop two minimal decision models for the exit choice. In the first one, the chosen exit is simply a function of the distance to the exits. In the second, the model depends on the distance to the exits and the density level in front of the exits. The models are based on linear discriminant analysis of the data.

### 4.1. Linear discriminant analysis

The first exit choice model is simply a function of the distance to the exits. The discriminant between two exits *i* and *j* is

4.1 $$Y_{1}=\gamma +\alpha {\tilde{d}}_{i}+\alpha_{0}{\tilde{d}}_{j}$$

${\tilde{d}}_{k}=d_{k}/d_{\max}$ being the relative distance to the exit *k*=*i*,*j*. In the second model, the exit choice depends not only on the distance but also on the density level in front of the exits The discriminant is

4.2 $$Y_{2}=\gamma +\alpha {\tilde{d}}_{i}+\alpha_{0}{\tilde{d}}_{j}+\beta {\tilde{\rho }}_{i}+\beta_{0}{\tilde{\rho }}_{j}$$

with ${\tilde{\rho }}_{k}=\rho_{k}/\rho_{\max}$ the relative density in front of the exit *k*=*i*,*j*. In this second model, the width of the area where the density is measured should depend on the width of the exit in order to measure a quantity proportional to the number of persons queuing in front of the exit.

We denote *Y* ^*i*^ the discriminant for the exit *i*. The parameter *P*=(*γ*,*α*,*α*_0_,*β*,*β*_0_) is estimated by minimizing the discriminant intra-variability $\sum_{i}\text{var}(Y^{i}(P))$. This corresponds to the maximization of the distance between the mean discriminants by exit. This estimation method is the maximum-likelihood one under homoscedasticity and multivariate normal distribution assumptions (e.g. [24]). The parameter estimations with $d_{\max}=12\;m$ and $\rho_{\max}=6\;\mathrm{ped}\;m^{-2}$ are given in table 1. The discriminant quantifies the exit choice as a function of distance and density. Negative values of the discriminant correspond to the choice of the first exit, while positive values correspond to the choice of the second exit. The sign of the estimations are reasonable : *α* (resp. *β*) is positive and *α*_0_ (resp. *β*_0_) is negative for the all the experiments (the distance (resp. the density) has a repulsive effect on the exit choice). More precisely, the values are approximately opposed while the constant *γ* is close to zero. Therefore, we do not detect bias such as preferred exit in the observations. Note that in order to keep the model as simple as possible, we only simultaneously compare two exits. In experiment B, which had four possible exits, we had to separately consider the chosen exit to one of the three others. Yet, the minimum operation used to select the exit is commutative, therefore, this approach does not change the nature of the model (the chosen exit is the one minimizing the discriminant, independently of the number of possible exits).

**Table 1.** Estimations of the parameters for the discriminant variables.

	*Y*_1_			*Y*_2_				
	*γ*	*α*	*α*_0_	*γ*	*α*	*α*_0_	*β*	*β*_0_
experiment A	−0.22	9.89	−9.01	−0.36	10.08	−9.06	1.61	−1.45
experiment B	0.23	2.88	−4.92	0.39	3.92	−5.56	7.09	−8.37
experiment C	0.27	8.95	−16.33	0.62	9.06	−16.75	2.6	−2.6
global sample	−0.23	4.91	−5.29	−0.1	5.68	−5.81	5.22	−4.41

**Figure 10.**
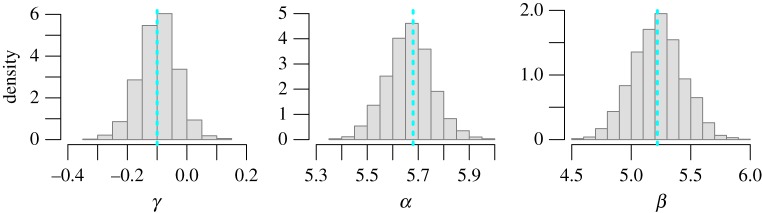
Histograms of the parameter estimations on sub-samples for the exit choice model based on the distance and the density with the global sample.

The parameter estimations differ from one sample to another. Yet, the values are close for experiment B and the global sample. The bootstrap method with 2000 sub-samples is used to evaluate the precision of the estimation. The histograms of the estimations are presented in figure 10. The variability is relatively low and the distributions of the parameters are symmetric. Therefore, the estimations can be considered precise.

**Figure 11.**
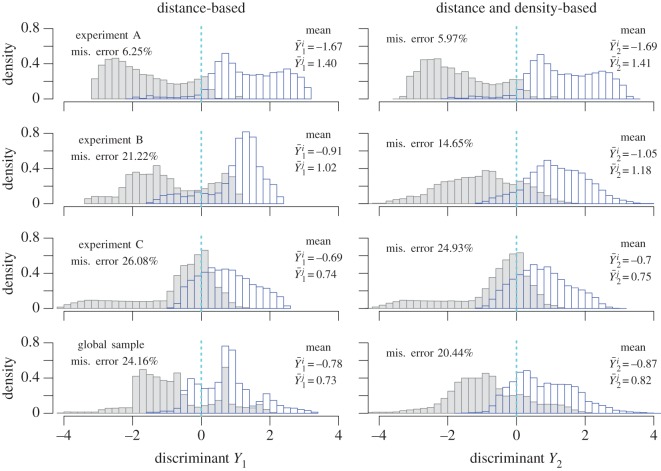
Histograms of the discriminant with mean values and misclassification error. The grey histogram corresponds to the discriminant for the chosen exit, while the blue histogram corresponds to the discriminant for one of the other exits.

The histograms of the discriminants are plotted in figure 11. The values tend to be negative for one exit and negative for the other. This means that the distance and the density allow (at least partially) to explain the exit choice. The misclassification error gives the percentage of wrong classification from the sign of the discriminant. The error decreases with the model based on the density. The Fisher test is significant for the experiments B and C, and the global sample (table 2). This confirms that the density has a role in the decision of the exit choice. More precisely, the distributions tend to be unimodal, more distinct and compact with the model depending on the density level in front of the exits. This is especially the case within experiment B and the global sample.

**Table 2.** Fisher test *p*-value for the comparison of the variance of the model solely based on the distance and the model based on the distance and the density. The variability is significantly reduced by adding the density in the decision model for the global sample and experiments B and C.

Fisher test *σ*_*Y*_1__=*σ*_*Y*_2__	experiment			global sample
	A	B	C	
*p*-value	2.50×10^−1^	1.11×10^−14^	1.82×10^−2^	<1×10^−22^

### 4.2. Decision model for the exit choice

The decision models for the exit choice are based on the results of the discriminant analysis. The estimations of the relative slope $\gamma /\bar{Y}$ of the discriminant are close to zero. Moreover, the coefficients for the distance and the density are approximately equal (at least for the global sample). The decision model is based on a roughly estimate of the discriminants. The exit choice model solely based on the distance is

$$\text{Choice of exit with minimal}\;{\tilde{d}}_{i},$$

while the exit choice model based on the distance and the density is

$$\text{Choice of exit with minimal}\;{\tilde{d}}_{i}+{\tilde{\rho }}_{i}.$$

Here, ${\tilde{d}}_{i}=d_{i}/d_{\max}$ is the relative distance to the exit *i* and ${\tilde{\rho }}_{i}=\rho_{i}/\rho_{\max}$ the relative density in front of the exit *i*. Note that the the first model is simply the shortest path. The parameters for the second model are $d_{\max}=12\;m$ and $\rho_{\max}=6\;\mathrm{ped}\;m^{-2}$.

## 5. Simulation results

Some simulations are performed to compare the exit choice model solely based on the distance to the exits, and the model based on the distance and the density level around the exits. The same settings as in the real experiments (i.e. same geometries and initial conditions) are used. The microscopic pedestrian models introduced in [25] are used to simulate the trajectories. For the distance-based model, the exit chosen is the closest one. For the distance- and density-based model, the exit is the one minimizing ${\tilde{d}}_{i}+{\tilde{\rho }}_{i}$. The exit choice is done at each time step of the simulation *δt*=0.1 *s*.

### 5.1. Experiment A

**Figure 12.**
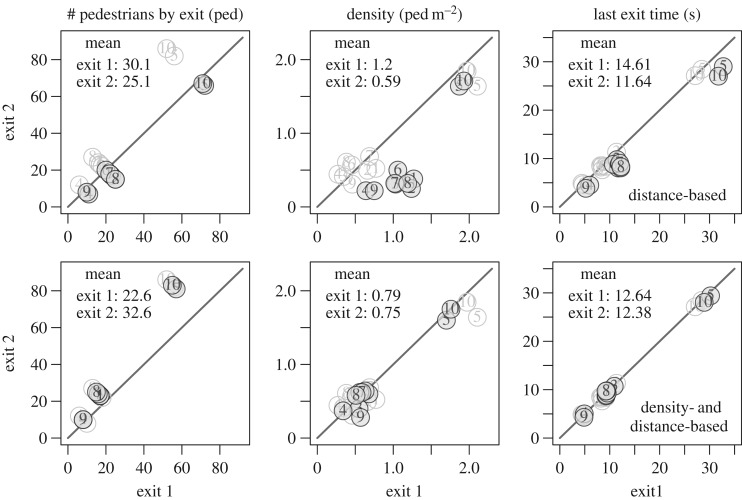
Experiment A—mean performances for the simulation results. The numbers identify the run. The black points are the simulation while the grey ones are the real data. The load balancing that is observed in the data does not occurs with the distance-based model (top panels). But arises with the density- and distance-based model (bottom panels).

The mean performances obtained for the experiment A are presented in figure 12. The black points correspond to the simulation while the grey ones are the real data. The numbers identify the run. In this experiment, the size of exit 1 is smaller than the size of exit 2. With the distance-based model, the pedestrians share equally the two exits, and more congestion occurs at the smallest exit (figure 12, top panels). Consequently, the last exit times are different: the load balancing does not occur. With the density and distance model, the pedestrians share as for the real data so that the densities and the last exit times are equal (load balancing) (figure 12, bottom panels).

### 5.2. Experiment B

**Figure 13.**
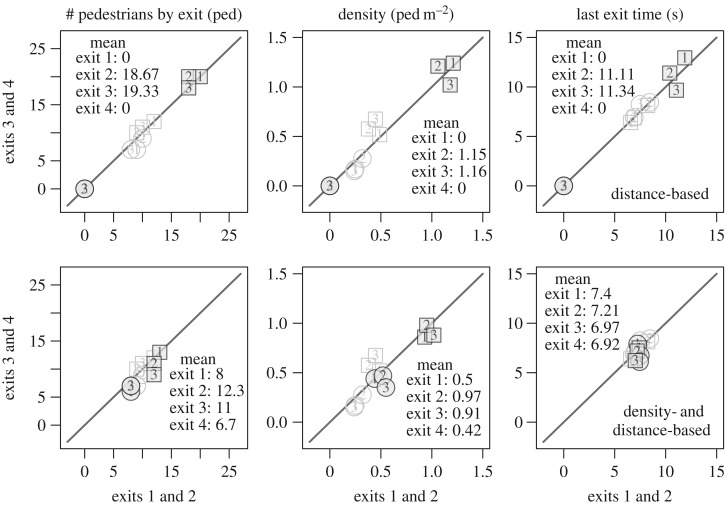
Experiment B—mean performances for the simulation results. The numbers identify the run. The black points are the simulations while the grey ones are the real data. The circles corresponds to exits 2 and 3 while the squares correspond to exits 1 and 4. The load balancing trivially does not occur with the shortest distance model because exits 1 and 4 are not used (top panels). It arises with the density- and distance-based model in good agreement to the data (bottom panels).

The mean performances obtained for the experiment B are presented in figure 13. Here, again the numbers identify the run. The black points correspond to the simulation while the grey ones are the real data. The circles correspond to exits 2 and 3 while the squares correspond to exits 1 and 4. Owing to the initial conditions, only exits 2 and 3 are used with the model solely based on the distance (figure 13, top panels). The load balancing clearly does not occur and the time needed to make the room empty is higher than the times observed in the real data. The results are improved with the model based on the density and the distance (figure 13, bottom panels). As observed, the two other exits 1 and 4 are also used and the load balancing occurs. Note that the density is lightly over-estimated with the model. This may overweight the role of the density in the exit choice model.

### 5.3. Experiment C

**Figure 14.**
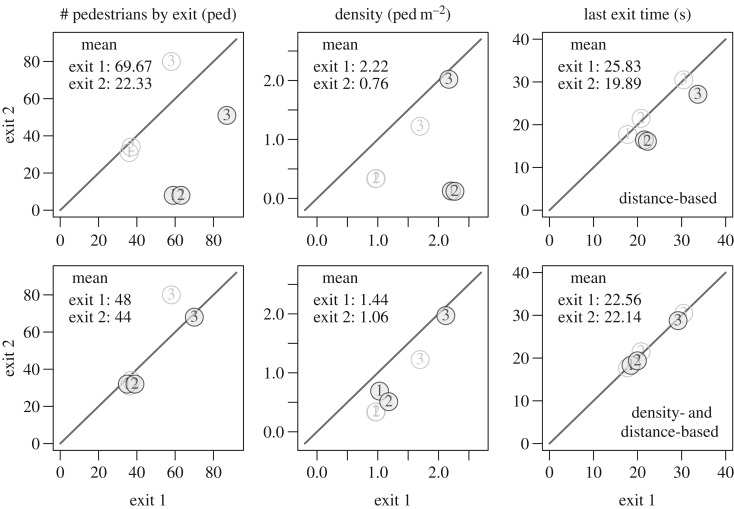
Experiment C—mean performances for the simulation results. The numbers identify the run. The black points are the simulations while the grey ones are the real data. Both exits are equitably used with the real data and the model based on the density (bottom panels) while the farthest exit is clearly underused with the model solely based on the distance (top panels).

The mean performances obtained for the experiment C are presented in figure 14. Here, again the load balancing occurs clearly with the density- and distance-based model (figure 14, bottom panels), and trivially not with the model solely based on the distance (figure 14, top panels). As a consequence the egress time is over-evaluated with the distance-based model.

## 6. Conclusion

New experiments about pedestrian exit choice in symmetric and asymmetric congested situations are presented. We observe a load balancing of the distribution of the participants over the exits for different initial conditions, resulting in optimization of the egress time. The results highlight that in a normal situation pedestrian exit choice is not solely based on the distance to the exits. Estimations of travel time taking into consideration the congestion is also preponderant.

Minimal exit choice models based on the distance to the exit and the density level in the vicinity of the exits are calibrated using a discriminant analysis. We observe that the use of the density significantly improves the model. These results are corroborated by simulation experiments. The model based on the distance describes unrealistic behaviours in the case of congestion or within asymmetric scenarios. The load balancing does not occur. The model including the density allows to describe the repartition of the pedestrians over the exits. The density, easy to estimate, substitutes the role of the travel time in the classical models. Yet, it provides robust dynamical optimization, as such observed in the data, without requirement of estimations of travel times.

This analysis allows to basically describe the exit choice of pedestrians in a normal situation and in the case of equivalent exits. Knowledge of the environment, preferences or again herding effects are not considered. Such phenomena require other mechanisms and parameters extending the minimal model we propose. The model only depends on the maximal distance $d_{\max}$ and density $\rho_{\max}$ parameters. The values used here for the different geometries are the same ($d_{\max}=12\;m$ and $\rho_{\max}=6\;\mathrm{ped}\;m^{-2}$). Further experiments regarding very crowded situations and the size of the measurement area for the density remain to be investigated. The model has been validated by simulation for three rooms, which configuration can be asymmetric, up to approximately 100 m^2^. In bigger rooms, some visibility constraints as well as nonlinear effects may make the model imprecise. For instance, a pedestrian may prefer to wait at a congested exit instead of going to a free exit farther just because, in the case of long distance, he/she wants to avoid to walk a long time. Finally, this minimal approach can lead in simulation to numerical oscillations related as the *ping–pong* effect. Such a drawback can be corrected by using inertial system and relaxation processes, and here again, additional parameters.

## Data accessibility

The data are freely available at http://ped.fz-juelich.de/database (video and extracted trajectories) and are deposited at the Dryad Digital Repository: http://dx.doi.org/10.5061/dryad.m5sd2 [26] (extracted trajectories).
